# Endothelin-1 and Vasomotor Tone Following Cardioplegic Ischemia/Reperfusion and Cardiopulmonary Bypass

**DOI:** 10.3390/cells15040346

**Published:** 2026-02-14

**Authors:** Shawn Kant, Frank Sellke, Jun Feng

**Affiliations:** 1 Department of Medicine and Warren Alpert Medical School, Rhode Island Hospital, Brown University, Providence, RI 02903, USA; shawn_kant@brown.edu; 2 Department of Cardiothoracic Surgery and Warren Alpert Medical School, Rhode Island Hospital, Brown University, Providence, RI 02903, USA; frank_sellke@brown.edu; 3 Department of Surgery, Heart Institute, Morsani School of Medicine, University of South Florida, Tampa, FL 33606, USA

**Keywords:** endothelin-1, cardiopulmonary bypass, ischemia–reperfusion, vasomotor tone, microcirculation, microvascular dysfunction

## Abstract

**Highlights:**

**What are the main findings?**

- Endothelin-1 is an important regulator of vasomotor tone in the human body
- Abnormal endothelin-1 activity can be observed in several tissue beds following cardiac surgery involving cardiopulmonary bypass, including the systemic, coronary, skeletal muscle, mesenteric, and pulmonary circulations

**What are the implications of the main findings?**

- Additional research is required to clarify some conflicting data regarding the nature of endothelin-1 dysfunction in certain vascular beds, such as in the coronary and skeletal microcirculations
- Aberrant endothelin-1 activity and signaling may be a potential therapeutic target for addressing postoperative vasomotor dysfunction following cardiac surgery involving cardiopulmonary bypass

**Abstract:**

Endothelin-1 is a potent regulator of vasomotor tone and promotes endothelium-dependent vasoconstriction of vascular smooth muscle. Dysregulated vasomotor tone is a hallmark of microvascular pathology following cardiac surgery involving cardioplegia and cardiopulmonary bypass (CPB). This review begins with a discussion of the molecular biology of endothelin-1, the structure and function of endothelin receptors, and an overview of endothelin signaling pathways and endogenous regulation. Following this, the focus will turn to an exploration of abnormal endothelin-1 activity during and after CPB across different vascular systems, including coronary, pulmonary, skeletal muscle, peripheral, and mesenteric circulation. Finally, this review concludes with a discussion of drugs targeting endothelin-1 signaling pathways to protect vasomotor tone and microvascular function from ischemia/reperfusion-induced damage, highlighting new therapeutic targets to reduce postoperative morbidity and mortality.

## 1. Introduction

The vascular endothelium plays a critical role in metabolic homeostasis, influencing multiple processes including vascular permeability, vascular tone, inflammation, and hemostasis. Endothelium-derived vasoactive mediators have an important role in preserving balance between vasoconstriction and vasodilation. Examples of endothelium-derived vasoconstrictors include endothelin-1 and thromboxane A2; endothelium-derived vasodilators include endothelium-derived hyperpolarizing factor, nitric oxide, and prostacyclin [1].

Endothelin-1 is a 21 amino acid peptide belonging to the endothelin family, first identified from porcine aortic endothelial cells in 1988 [2,3]. Since then, a flurry of studies have shown that endothelin-1 is a potent vasoconstrictor and major regulator of vasomotor tone in vascular beds throughout the body [4,5,6]. However, with time, endothelin-1 has been shown to affect far more than just vasomotor tone. Indeed, endothelin-1 has been implicated in renal hemodynamics, mitogenesis, energy metabolism, inflammation, bone homeostasis, and a variety of other metabolic processes, testifying to its immense physiologic significance and the need for comprehensive understanding of its biology [6,7]. Importantly, there are even data suggesting that preoperative endothelin-1 levels can be an independent metric of postoperative morbidity and mortality for patients undergoing cardiac surgery involving cardiopulmonary bypass (CPB) [8,9].

In this review, we first discuss the molecular biology of endothelin-1, its receptors, and signaling pathways. Then, we proceed into a detailed survey of the current literature concerning abnormal endothelin-1 activity as it pertains to vasomotor dysfunction due to ischemia–reperfusion during cardiac surgery involving CPB. Importantly, we focus our survey on animal models and adult patients undergoing cardiac surgery involving CPB, forgoing discussion of infants/children undergoing cardiac surgeries involving CPB for congenital malformations, which would be a worthy topic for a separate review of its own given fundamental differences between adult and infant cardiovascular physiology.

## 2. Endothelin-1 Biology, Pathways, and Regulation

### 2.1. Molecular Biology of Endothelin-1

**Figure 1:**
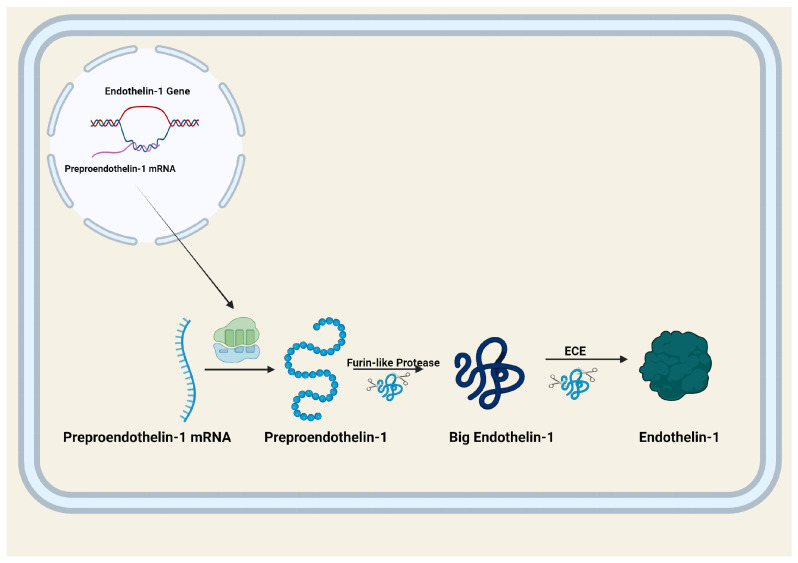
Endothelin-1 synthesis. The gene for endothelin-1 is transcribed into preoproendothelin-1 mRNA in the nucleus, after which it exits the nucleus and is translated into preproendothelin-1. Preproendothelin-1 is cleaved by furin-like protease into big endothelin-1. Endothelin converting enzyme (ECE) cleaves big endothelin-1 into mature endothelin-1. Figure created using biorender.com; https://www.biorender.com/.

Endothelin-1 synthesis begins with translation of mRNA derived from the Edn1 gene of chromosome 6 into preproendothelin-1, a 212 amino acid precursor protein [7]. Edn1 spans about 6.8 kb of genomic DNA, and contains 5 exons (of note, for additional background, there are three distinct endothelin peptides produced by mammalian species including humans—endothelin-1, endothelin-2, and endothelin-3, all of which have their own distinct biological activity profiles [3]). Preproendothelin-1 is then processed in a sequence of proteolytic steps into mature endothelin-1 [7]. These steps include cleavage of preproendothelin-1 into big endothelin-1, an inactive intermediate, which can then undergo two different paths to becoming mature endothelin-1. Big endothelin-1 may be cleaved by endothelin-converting enzyme (ECE) directly into endothelin-1, or by chymase into endothelin-1 (1–31), which is then cleaved by neprilysin in a two-step process [10,11,12]. Figure 1 depicts major steps in the process of endothelin-1 biosynthesis.

### 2.2. Endothelin-1 Receptor Structure

There are two broad types of endothelin receptors, ETA and ETB, both of which are class A G protein-coupled receptors (GPCR) [13,14,15,16,17]. All three endothelin peptides (endothelin-1, 2, and 3) act on both ETA and ETB, with varying degrees of affinity. Indeed, ETA has a 100-fold stronger affinity for endothelin-1 and 2 than it does for endothelin-3 [18,19]. Meanwhile, ETB has roughly equal affinity for all three endothelins [18,19]. Even more interesting, although endothelins are best known for being potent vasoconstrictors, ETA and ETB have opposing effects on vasomotor tone: ETA promotes long-acting vasoconstriction, while ETB mediates vasodilation [20,21].

**Figure 2:**
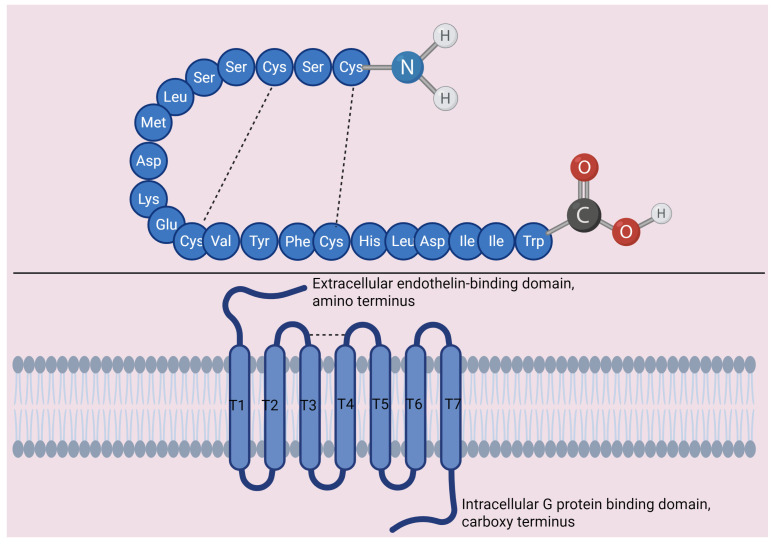
Endothelin 1 and receptor structure. Top: peptide structure of endothelin-1. Note presence of cysteine bonds (dashed lines). Cys = cysteine, ser = serine, leu = leucine, met = methionine, asp = aspartate, lys = lysine, glu = glutamate, val = valine, tyr = tyrosline, phe = phenylalanine, his = histidine, ile = isoleucine, trp = tryptophan. Bottom: general structure of endothelin receptors, applies to ETA and ETB. Both are G protein-coupled receptors with seven transmembrane domains (T1-T7). Endothelin binds to an extracellular binding domain near the N terminus. Intracellularly, the receptors associate with G proteins at the C terminus. Figure created using biorender.com; https://www.biorender.com/.

As is true for most classical GPCRs, ETA and ETB both consist of seven key alpha helical transmembrane domains, three extracellular loops, and three intracellular loops, and depend on recruitment of G proteins upon activation [13]. Specific amino acid residues of endothelin peptides interact with a large-scale outward kink at the extracellular end of transmembrane domain 5 [13]. Moreover, the c-terminal region of endothelin-1 can further associate with the ETA/ETB receptor cores via hydrogen bonding and hydrophobic interactions with residues in the core cavity [13,22]. A disulfide bond caps the peptide-binding pocket of ETA and ETB by linking the N-terminus of the receptor and the extracellular end of transmembrane domain 7 [13]. This latter feature may facilitate long-lasting activation of the ETA and ETB receptors by increasing the duration of endothelin binding with the receptor. Figure 2 shows a general schematic of endothelin-1 and receptor structure.

### 2.3. Localization of Endothelin-1 and Endothelin Receptors

Endothelin-1 is primarily produced by vascular endothelial cells, although it has a fairly wide distribution throughout the body. Indeed, beyond vascular endothelial cells, endothelin-1 can be found in high concentrations in the adrenal cortex, placenta, neurons, lung parenchyma, osteoblasts and osteoclasts, and fallopian tubes [23,24,25]. With respect to endothelin receptors, ETA and ETB are both found across the cardiovascular system, in coronary arteries, subcutaneous arteries, pulmonary artery, mammary artery and veins, glomerular capillaries, cardiomyocytes, airway smooth muscle, osteoblasts, neurons, and vascular adventitial fibroblasts [26]. ETA receptors may also be found in adipocytes and hepatic stellate cells [26,27]. ETB receptors may also be found in renal tubules, hepatocytes, and endocrine tissue [26,28]. See Table 1 for a summary.

### 2.4. Regulation of Vascular Endothelin-1 Gene Expression

A variety of molecular signaling pathways have been shown to modulate vascular endothelin-1 gene expression. One pathway is insulin signaling. Insulin inactivates glycogen synthase kinase 3 beta via activation of phosphoinositide-3 kinase (PI3K), in turn relieving inhibition of the transcription factor vascular endothelial zinc finger-1, which is required for edn1 gene promotor activity and transcription [29]. This process gone awry may contribute to increased propensity to microvascular dysfunction due to abnormally high endothelin-1 production in hyperinsulinemic and insulin-resistance states, such as diabetes.

Protein kinase C (PKC) is another regulator of endothelin-1 gene expression through activation of AP-1, another transcription factor that influences the edn1 gene promotor [30,31,32,33]. PKC also appears to serve as a final common pathway through which other molecules regulate endothelin-1 gene expression, such as angiotensin II and vasopressin, high-density lipoproteins, and thrombin [34,35,36]. Excessive endothelin-1 activity in a setting of protein kinase C hyperactivity has been implicated in a variety of vascular disease states characterized by abnormal microvascular tone and microvascular inflammation, including diabetes, hypertension, ischemia–reperfusion, and atherosclerosis [33,37]. Further testing of the AP-1 binding site at the edn1 promotor has identified even more modulators of edn1 expression. These include PPARgamma agonists and homocysteine, which block AP-1 binding to the edn1 promotor and thereby inhibit endothelin-1 expression [38,39].

Another potent regulator of vascular endothelin-1 expression is hypoxia, and hypoxia inducible factor-1 (HIF-1) [40]. HIF-1 alpha and HIF-1 beta both bind to the edn1 promotor and influence hypoxia-dependent stimulation of endothelin-1 expression [41]. Some studies suggest that tyrosine kinases and PI3K may play a role in hypoxic regulation of edn1. Zhang et al. showed that application of tyrosine kinase and PI3K inhibitors abolished hypoxia-induced increased preproendothelin-1 mRNA levels [37]. Meanwhile, Michenko and Caro showed that tyrosine kinase inhibition appears to affect transcriptional activity of the HIF-1 complex without affecting its DNA binding properties, nonetheless resulting in decreased endothelin-1 expression [42]. Of note, ethanol also induces HIF-1 alpha expression, increased HIF-1 alpha binding to edn1, and increased endothelin-1 expression in liver sinusoidal endothelial cells, which may facilitate inflammation and alcohol-induced liver injury [43].

Transforming growth factor beta (TGFbeta) is a potent inducer of endothelin-1 expression via Smad protein activity at the edn1 promotor [44]. TGFbeta accelerates recruitment of Smad3 and Smad4 to the edn1 promotor of vascular endothelial cells through activation of endothelial activin receptor-like kinase 5 [45]. Smad3 and AP-1 (discussed above) together activate edn1 gene expression in response to TGFbeta, with p300 possibly serving as a bridge between Smad3 and AP-1, although the latter is somewhat poorly defined and requires more elucidation [7].

Aldosterone has been shown to stimulate edn1 expression in vascular smooth muscle cells, with endothelin-1 augmenting the effects of aldosterone on blood pressure and sodium homeostasis by promoting the transcription of serum- and glucocorticoid-regulated kinase-1 mRNA [46]. Mineralocorticoid and glucocorticoid receptors bind to the edn1 promotor in an aldosterone-dependent fashion [7]. The relationship may also be reciprocal in the sense that endothelin-1 (1–31) may itself promote growth of the adrenal cortex and stimulation of aldosterone secretion in a feedback loop [47].

Furthermore, the edn1 promotor contains an NF-kB binding site, pointing to a role for NF-kB in edn1 expression regulation. NF-kB binding to the edn1 promotor in bovine aortic endothelial cells showed increased endothelin-1 mRNA transcription, with overexpression of cytoplasmic inhibitor of NF-kB or deletion of NF-kB binding site on edn1 mitigating against endothelin-1 transcription [48]. Likewise, certain cytokines such as tumor necrosis factor alpha (TNF-alpha) modulate the expression of endothelin-1. TNF-alpha induces release of endothelin-1 from bovine aortic endothelial cells in a time and concentration-dependent manner in conjunction with augmented preproendothelin-1 mRNA transcript levels [49].

This appears to occur through TNF-alpha-mediated induction of AP-1 and NF-kB and their subsequent actions on the edn1 gene promotor, along with increased activation of c-Jun NH2-terminal kinase and p38 mitogen-activated protein kinase 9 activity [50]. With respect to NF-kB, TNF-alpha induced degradation of its inhibitor IkBalpha [50]. Similarly, isolated small arteries from obese patients showed a link between increased TNF-alpha levels and endothelin receptor ETA and ETB expression, which was blunted with infliximab treatment [51].

Turbulent blood flow and vascular wall shear stress affect endothelin 1 production in vascular endothelial cells, although the specific effect is quite complicated and appears to depend on the duration and level of shear stress. Based on studies involving human umbilical vein endothelial cells, brief exposure (e.g., <1 h) or low levels (e.g., 1.8 dyn/cm^2^) of shear stress trigger sustained stimulation of endothelin-1 release, which may be blocked by PKC inhibition [52,53,54].

Indeed, others have shown that mechanical strain due to negative pressure deformation of vessel walls produced increased endothelin-1 mRNA levels, with PKC inhibition abolishing this effect [55]. However, sustained exposure to shear stress (e.g., >6 h) or intense shear stress (e.g., 6–25 dyn/cm^2^) resulted in downregulation of preproendothelin-1 mRNA in a dose-dependent manner, along with downregulation of ECE-1a and ECE-1b mRNA [52,53]. Curiously, this was not affected by PKC or tyrosine kinase inhibition but was affected by endothelial nitric oxide synthase inhibition, which prevented preproendothelin-1 downregulation [52,53].

### 2.5. Endothelin-1 Signaling Pathways

**Figure 3:**
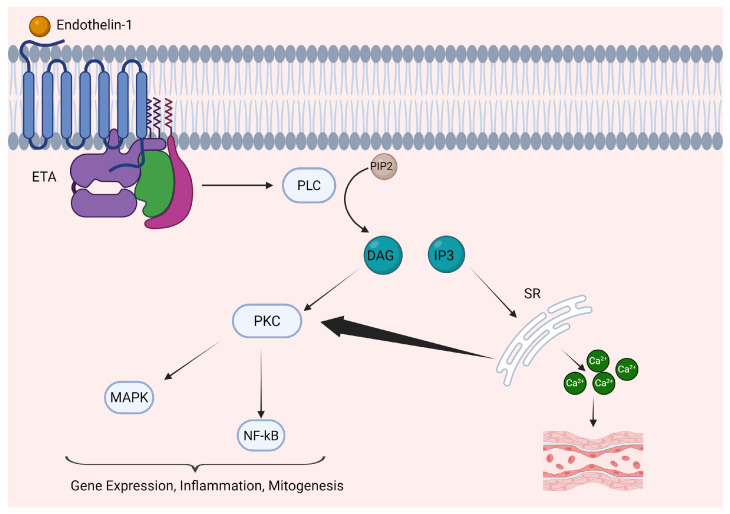
Endothelin-1 signaling pathway in vascular smooth muscle cells. Endothelin-1 binds to the ETA receptor shown here, a G-protein coupled receptor. This leads to activation of phospholipase C (PLC), which cleaves PIP2 to IP3 and diacylglycerol (DAG). IP3 facilitates calcium release from smooth endoplasmic reticulum stores (SR). Calcium in turn facilitates vascular smooth muscle contraction, and also acts in concert with DAG to activate protein kinase C (PKC). PKC acts through several pathways, including those involving MAPK, NF-kB, and other transcription factors, to influence gene expression, inflammation, and mitogenesis among other key homeostatic processes. Figure created using biorender.com; https://www.biorender.com/.

Figure 3 depicts a classical endothelin-1 signaling pathway. Agonist binding to ETA and ETB leads to rearrangement of several downstream conserved motifs in the receptor, causing several conformational changes. These include rotation towards transmembrane domain 3 and inward movement of transmembrane domain 7, resulting in tighter packing against transmembrane domain 3 [56]. Ultimately, the activation signal leads to displacement of the cytoplasmic end of transmembrane domain 6, facilitating G protein recruitment on the cytoplasmic side of the GPCR’s helices [57].

The canonical pathway of endothelin-1 signaling goes as follows. Activated G proteins from ETA and ETB stimulate activity of phospholipase C, which converts phosphatidylinositol 4,5-bisphosphate (PIP2) to inositol trisphosphate (IP3) and diacylglycerol (DAG) [58,59]. The ETA receptor may also trigger activation of phospholipase D, which hydrolyzes phosphatidylcholine [58]. IP3 triggers calcium release from intracellular stores such as those in the sarcoplasmic reticulum or smooth endoplasmic reticulum, while DAG in conjunction with calcium activate protein kinase C, a major mediator of multiple intracellular processes involving inflammation, mitogenesis, metabolism, and regulation of vasomotor tone [33]. Further downstream effects may also involve activation of calcium-permeable non-selective cation channels, and stimulation of extracellular signal-regulated kinases (ERK) [60].

Depending on the location of the endothelin receptor, there are slight variations in the results of these downstream signaling pathways. In blood vessels, which are the focus of this review, the primary effects of endothelial and vascular smooth muscle ETA receptors, as well as vascular smooth muscle ETB receptors, are constriction of vascular smooth muscle and vascular remodeling [26,61]. However, vascular endothelial ETB promotes nitric oxide and prostacyclin PGI2 production, which promotes vascular smooth muscle relaxation [26]. Elevated intracellular calcium may activate endothelial nitric oxide synthase, along with COX-2 (prostacyclin synthesis from arachidonic acid metabolism), and facilitates opening of small-conductance calcium-activated potassium channels (SK channels) [26,62]. Nitric oxide promotes activation of guanylyl cyclase, and PGI2 promotes activation of adenylyl cyclase, generating cGMP and cAMP respectively, both of which facilitate vasodilation in conjunction with potassium efflux through SK channels [26,62].

## 3. Abnormal Endothelin-1 Activity Following Cardioplegic Ischemia-Reperfusion

### 3.1. Background

Oxygen deprivation imposes significant deleterious consequences on all major organ systems, as all systems require oxygen to sustain aerobic metabolism. While some organs may sustain themselves for potentially hours or longer in hypoxic or ischemic conditions (e.g., skeletal muscle), others, such as the heart, lungs, and brain, sustain severe damage within minutes of oxygen deprivation. Hypoxic/ischemic injury often occurs in disease states characterized by micro and macrovascular dysfunction, including thromboembolism (e.g., acute stroke, pulmonary embolism, myocardial infarction), diabetes, atherosclerosis, and abnormal vasomotor and myogenic tone. It may also occur during certain medical procedures, notably during cardiac surgery involving cardiopulmonary bypass [63,64,65].

Even as minimally invasive procedures, such as percutaneous coronary interventions and transcatheter aortic valve replacements, become ever more popular alternatives to major cardiac surgeries, for the present time surgical interventions such as coronary artery bypass grafting (CABG) and surgical aortic and mitral valve replacements remain crucial mainstays of cardiovascular medicine. Indeed, about 400,000 CABG surgeries are performed in the US on average annually, with the majority done under CPB [66,67].

While there are many different nuances and techniques to CPB, mostly involving the temperature and chemical composition of cardioplegic solutions, the general concept is as follows [63,68]. In essence, CPB diverts blood from the heart and lungs through venous cannulas [63,68]. Blood then moves through the external CPB circuit, which provides oxygenation, electrolyte adjustment, and thermoregulation [63,68]. Finally, blood returns to the patient’s circulation through arterial cannulas [63,68]. The end result is complete emptying of the heart and heart-independent perfusion of vital organs to allow the surgeon to operate on a stilled heart.

Despite major advances in cardioprotective strategies over the years, ischemia–reperfusion injury remains a significant postoperative problem following CPB, often manifesting as microvascular endothelial dysfunction and impaired myocardial and systemic organ perfusion [69,70,71]. CPB also induces a systemic inflammatory response via a combination of pro-inflammatory cytokine release, leukocyte activation, and oxidative stress as blood moves through the CPB circuit [72]. Moreover, deep hypothermic cardioplegia may also deplete myocardial antioxidants, such as glutathione, which further exacerbates perioperative oxidative stress and myocardial membrane lipid peroxidation [73].

All of these effects likely contribute to vasoplegic syndrome, a frequently described state following cardiac surgery involving CPB consisting of form of distributive shock characterized by hypotension refractory to fluid challenges, systemic vascular resistance < 800 dynes units, and a cardiac index greater than 2.2 [74,75]. Key molecular features of vasoplegic syndrome are as follows. First, exposure of blood to the CPB circuit and reperfusion-induced reactive oxygen species generation triggers cytokine storm (predominantly IL-1, IL-6, and TNF-alpha), leading to inducible nitric oxide synthase activation and nitric oxide generation [74,76]. Through activation of guanylyl cyclase, nitric oxide increases cyclic GMP production and promotes opening of K-ATP and SK channels, leading to vascular smooth muscle hyperpolarization [74]. Likewise, CPB promotes desensitization of adrenergic receptors and impaired responses to potent vasoconstrictors such as vasopressin, norepinephrine, and angiotensin 2. Risk factors for vasoplegic syndrome include low preoperative left ventricular ejection fraction, advanced age, prolonged aortic cross-clamp and CPB time [74,76].

A growing body of literature suggests that endothelin-1 has a role in mediating endothelial and vascular smooth muscle dysfunction due to hypoxia/ischemia–reperfusion injury. First, there is strong evidence that endothelin-1 promotes NADPH oxidase activation, and therefore stimulates production of superoxide and other reactive oxygen species in cardiomyocytes and vascular cells [77,78,79]. Likewise, reactive oxygen species themselves enhance endothelin-1 mRNA expression, therein creating a pathologic feedback loop that amplifies oxidative stress, which, as discussed above, is a critical driver of hypoxia/ischemia–reperfusion injury (e.g., via interference with nitric oxide mediate coronary vasodilation, promotion of apoptosis, altered calcium homeostasis, etc.) [80,81,82,83]. Furthermore, downstream activation of NF-kB, ERK ½, Akt, and bcl-2 related pathways by endothelin-1 augment pathologic microvascular remodeling and creation of a pro-inflammatory metabolic cellular microenvironment [84].

Once can easily see the numerous parallels between all of these effects and the established molecular basis of microvascular dysfunction following CPB. Hence, the focus of the remainder of this review is to survey the state of the current literature regarding aberrations in endothelin-1 activity and signaling following hypoxia/ischemia–reperfusion injury in the context of CPB.

### 3.2. Perioperative Endothelin-1 Levels During and After CP/CPB: Basic Principles, Techniques of CPB, and Systemic Circulation Studies

Beginning with the systemic circulation, a variety of studies show that CPB has a significant effect on perioperative endothelin-1 levels in arterial and venous systems. First, Goodwin et al. showed that isolated rat hearts perfused with Krebs buffer subjected to 4 h of cardioplegic arrest followed by 2 h of reperfusion exhibited increased ECE mRNA expression without corresponding increases in preproendothelin-1 mRNA expression [85]. Walker et al. found a significant increase in systemic endothelin-1 levels post-CPB in a pig model [86].

Similar results were noted in several studies involving human patients undergoing CABG while on CPB, with postoperative systemic arterial endothelin-1 levels significantly increased relative to preoperative levels, by a magnitude of up to 85% [87,88,89,90,91,92,93]. Bond et al. took this one step further and observed increased internal thoracic artery sensitivity to endothelin-1 in patients requiring prolonged postoperative vasodilator support following on-pump CABG [89]. These effects may be magnified in patients with pre-existing metabolic diseases such as diabetes mellitus, an observation that requires further investigation to better elucidate mechanisms [94,95].

Furthermore, endothelin-1 activity is heightened in saphenous veins harvested during cardiac surgery involving CPB, and in vitro studies have connected ETA and ETB hyperactivity with increased saphenous vein vascular smooth muscle proliferation and neointimal thickening [96,97]. In fact, excessive endothelin-1 activity has even been tied to graft failure following CABG [97].

Importantly, different techniques of cardiac surgery and CPB appear to have an influence on perioperative endothelin-1 levels. First, patients undergoing off-pump CABG exhibited far less significant increases in systemic arterial and venous endothelin-1 levels than patients undergoing CABG involving CPB [88,90,98]. This is not too surprising, considering the extensive pro-inflammatory effects of the CPB circuit at the cellular and molecular levels, as discussed above. Next, pulsatile CPB resulted in significantly lower postoperative systemic endothelin-1, IL-8, epinephrine, and norepinephrine levels than nonpulsatile/continuous CPB in human patients [99].

A substantial body of research shows that heparin-coated bypass circuits reduces the extent of complement and granulocyte activation triggered by blood coming in contact with the CPB circuit during cardiac surgery [100,101]. This is evidenced by parameters such as decreased maximum levels of complement components C3b, iC3b, C3c, and terminal C5b-9 complex levels in patients undergoing CPB with heparin-coated circuits relative to standard circuits, along with lower maximum granulocyte myeloperoxidase and lactoferrin levels [100]. Extending this concept, Lundblad et al. found that while CPB increased overall systemic plasma endothelin-1 levels in all patients undergoing CPB, patients who underwent CPB with heparin-coated circuits had lower magnitudes of increased perioperative endothelin-1 levels relative to patients who underwent CPB without heparin-coated circuits [102].

The role of CPB temperature in endothelin-1 dynamics is complicated. For example, some studies have shown that hypothermic CPB (28 degrees Celsius) resulted in sustained higher systemic endothelin-1 levels postop compared to normothermic CPB (37 degrees Celsius) in patients undergoing CABG [103]. The exact mechanism of this observation is unclear; however, potential theories include cold cardioplegia-induced displacement of endothelin-1 from binding sites, increased ECE activity, or even downstream posttranslational effects in the endothelin-1 signaling pathway [103,104]. However, Yamada et al. found that while hypothermic CPB resulted in impaired endothelial function, the degree of hypothermia (ranging from 18 to 34 degrees Celsius) had no effect on plasma endothelin-1 levels [105]. Additional studies will be required to clarify the impact of cardioplegia/CPB temperature on systemic endothelin-1 levels.

### 3.3. Endothelin-1 and Skeletal Muscle Vasoconstriction Following CP/CPB

Turning to specific vascular beds, Feng et al. found that post-CPB contractile responses of human coronary and skeletal muscle arterioles to endothelin-1 were significantly decreased relative to pre-CPB, pointing to a possible contribution of poor endothelin-1 responses towards vasoplegic syndrome post-CPB in these vascular beds [106,107]. Of note, these effects were more pronounced in diabetic patients [92]. Treatment with ETA receptor antagonists further inhibited skeletal muscle arteriolar responses to endothelin-1, while ETB receptor antagonists had no effect [106,107]. Finally, pretreatment with the PKC-alpha inhibitor salfingol reversed endothelin-1 induced responses from vasoconstriction to vasodilation, testifying to the role of PKC in endothelin signaling discussed earlier [106]. Curiously, the authors observed no changes in total protein expression levels of ETA, ETB, or endothelin-1 gene expression, suggesting that these effects are likely post-translational [106].

### 3.4. Endothelin-1 and Mesenteric Vasoconstriction Following CP/CPB

We briefly touch on mesenteric circulation, although the literature here is quite scant. High serum levels of endothelin-1 have been associated with mesenteric ischemia, and human cohort studies have shown that elevated endothelin-1 serum levels were predictive of nonocclusive mesenteric ischemia to a high degree of accuracy [108]. With respect to CPB, pig models of CPB exhibit increased levels of endothelin-1 in jejunal microvessels following CPB [109].

### 3.5. Endothelin-1 and Coronary Vasoconstriction Following CP/CPB

Moving to coronary circulation, the data regarding the effect of CPB on endothelin-1 levels and activity are somewhat mixed. First, Unic et al. showed that post-CBP endothelin-1 levels in the coronary circulation were significantly higher than pre-CPB [110]. Coronary endothelin-1 levels also correlated positively with 24-h-postoperative troponin-I levels, suggesting that postoperative endothelin-1 may have value as a biomarker of myocardial strain/injury in its own right. Verma et al. studied coronary sinus and atrial microvascular endothelin-1 levels at baseline and after CPB, and found significantly increased endothelin-1 mediated vasoconstriction [111]. ETA antagonism blocked these effects [111].

However, other studies show opposite findings. For example, Feng et al. found decreased, not increased, coronary arteriolar contractile responses to endothelin-1 following CPB in conjunction with no observed changes in protein expression of ETA and ETB [112]. Only ETA receptor antagonists had any effect on coronary arteriolar endothelin-1 responses [112]. Further work will be needed to reconcile these opposing findings and better elucidate the role of endothelin-1 as it pertains to postoperative vasoplegia and vasospasm in the coronary microcirculation following CPB.

More recently, our group showed that coronary microvascular contractile responses to endothelin-1 in patients with uncontrolled hypertension were increased compared to patients with controlled hypertension and normotensive patients at baseline [113]. These findings persisted post-CP/CPB, although post-CP/CPB microvascular responses to ET-1 were blunted overall [98]. ETA receptor antagonism diminished contractile responses in all groups [113]. Transcriptomics and proteomics showed wide regulation of pro-vasoconstrictive pathways [113]. Furthermore, patient hypertension status and cardiac mass predicted coronary microvascular contractile response to ET-1, which, in turn, predicted postoperative changes in ejection fraction [113].

### 3.6. Endothelin-1 and Pulmonary Vasoconstriction Following CP/CPB

Moving forward, numerous studies have examined endothelin-1 in the pulmonary circulation following CPB. First, animal models: porcine models of cardioplegic arrest and CPB exhibited significantly increased pulmonary vascular resistance and postoperative pulmonary hypertension, signs of increased pulmonary arteriolar vasoconstriction [114,115,116]. With respect to endothelin-1 specifically, pig pulmonary arteries exhibited increased contractile responses to endothelin-1 after CPB, and administration of ETA/ETB receptor antagonist bosentan prior to CPB had a protective effect against postoperative pulmonary hypertension [114,116].

Human studies of the pulmonary circulation before and after CPB largely track with animal models. Indeed, Unic et al. found significantly elevated levels of endothelin-1 following CPB in the human pulmonary circulation compared to pre-bypass baselines, similar to Kirshbom’s pig model [111,115]. Meanwhile, Bond et al. showed that human pulmonary arterial endothelin levels increased 50–85% following on-pump CABG [89]. However, we do note that some studies found no significant differences in pulmonary artery endothelin-1 levels pre- and post-CPB [90,117]. Therefore, future studies will need to clarify the impact of CPB on perioperative pulmonary arterial endothelin-1 dynamics. Table 2 summaries key changes in endothelin-1 activity following CPB in different vascular beds discussed in this section.

## 4. Abnormal Endothelin-1 Signaling in CP/CPB: A Therapeutic Target?

To conclude this discussion of endothelin-1 and ischemia/reperfusion injury in the setting of CPB, we present a series of studies testing interference with endothelin pathways as potential therapeutic tools for mitigating postoperative microvascular and organ dysfunction. First, application of endothelin receptor antagonists, in particular ETA blockade, significantly attenuates increases in pulmonary vascular resistance after CPB in pig and lamb models, without significant changes in systemic perfusion pressures [118,119,120,121]. Here, it is also worth highlighting that Petrossian et al. observed a direct positive correlation between post-CPB pulmonary vascular resistance and enothelin-1 concentration [120]. Likewise, Pearl et al. found that piglets treated with bosentan during the period of hypoxia exhibited stable arterial nitrite levels during hypoxia, unlike controls (which exhibited decreased arterial nitrite levels) [121]. Bosentan-treated piglets also displayed better post-CPB A-a gradients and lower postoperative myeloperoxidase levels, signifying improved ventilation/perfusion matching along with lower perioperative inflammation [121].

Moving beyond direct endothelin receptor antagonism, Kirshbom et al. found that pretreating pigs with the endothelin-converting enzyme (ECE) inhibitor phosphoramindon also protected against increased pulmonary vascular resistance following CPB [122]. Calpain inhibition also blunted endothelin-1 responses, decreased plasma endothelin-1 levels, and blocked increased pulmonary vascular resistance in a pig model of CPB [123]. Moreover, in yet another pig model of CPB, administration of pre- and intraoperative methylprednisolone decreased endothelin-1 levels following CPB and blocked increased pulmonary vascular resistance; curiously, the protective effect of glucocorticoids required both pre- and intraoperative treatment, as intraoperative treatment alone had no significant effect [124].

Human studies of endothelin antagonism have shown similar results in the pulmonary circulation. For example, Ikonomidis et al. found that perioperative treatment with ETA antagonists produced a dose-dependent reduction in pulmonary arterial pressure elevation following CPB, with no adverse effects directly attributable to drug treatment [125]. Toole et al. had similar results: treating patients undergoing CPB with pre-existing left ventricular dysfunction with ETA antagonist sitaxsentan immediately before separation from CPB and 12 h post-CPB protected against increased pulmonary vascular resistance following surgery [126].

Inspired oxygen fraction after CPB may have an effect on endothelin-1 activity in the systemic circulation along with pulmonary gas exchange. Indeed, patients whose lungs were ventilated with FiO2 of 1.0 after CPB weaning exhibited greater arterial and mixed venous big endothelin-1 concentrations than patients ventilated with FiO2 0.35, even as endothelin-1 concentrations in both groups were not significantly different—although both increased relative to preprocedure levels (Reber et al., 2000) [127]. Moreover, patients ventilated with higher FiO2 postoperatively had greater venous admixture than patients ventilated with lower FiO2 concentrations [127].

## 5. Conclusions

Endothelin-1 is an instrumental modulator of vascular tone in microcirculations throughout the body through its actions on vascular endothelial ETA and ETB receptors. Activation of ETA and ETB triggers potent calcium and PKC-mediated vasoconstriction, and a variety of factors regulate endothelin-1 signaling, including PKC itself, HIF-1 alpha, NF-kB, insulin, TGF-beta, TNF-alpha, and microvascular shear stresses. Abnormal endothelin-1 signaling, as occurs during and after CPB, contributes to significant microvascular dysfunction. This takes different forms in different vascular beds, such as reduced endothelin-1-mediated contractile responses in the skeletal circulation, increased endothelin-1-mediated contractile responses in the pulmonary circulation, and mixed effects in the coronary circulation. Meanwhile, endothelin-1 signaling poses an attractive therapeutic target for developing new drugs aimed at treating postoperative microvascular dysfunction, vasoplegia, and vasospasm following CPB, via tools such as ETA/ETB receptor blockade and ECE inhibition. Future studies will need to expand on this work to better characterize aberrant endothelin-1 signaling in specific organ microcirculations, in particular the coronary circulation where the data is somewhat mixed, and further investigate the safety and efficacy profiles of endothelin-1 pathway modulators in animal and human models.

## Figures and Tables

**Table 1:** Localization of endothelin-1 receptors.

ETA Only	ETB Only	ETA and ETB
Adipocytes	Renal tubular cells	Coronary arteries
Hepatic stellate cells	Hepatocytes Endocrine tissues	Subcutaneous arteries Pulmonary arteries Mammary arteries and veins Glomerular capillaries Cardiomyocytes Airway smooth muscle Osteoblasts Neurons

**Table 2:** Endothelin-1 activity following CPB in different vascular beds.

Location	Model	Observed Effect of CPB on Endothelin-1 Activity	Reference
Systemic Circulation (arterial and venous)	Pig	Increased plasma endothelin-1 levels	[86]
Systemic Circulation (arterial and venous)	Humans	Increased plasma endothelin-1 levels	[87,88,89,90,91,92]
Skeletal Muscle Arterioles	Humans	Decreased endothelin-1 mediated constriction	[106,107]
Mesenteric Circulation	Pig	Increased mesenteric endothelin-1 levels	[109]
Coronary Circulation	Human	Increased endothelin-1 levels	[110,111]
Coronary Circulation	Human	Decreased coronary endothelin-1 mediated contraction	[112,113]
Pulmonary Circulation	Pig	Increased endothelin-1 contractile responses	[114,116]
Pulmonary Circulation	Human	Increased plasma endothelin-1	[110,115]
Pulmonary Circulation	Human	No significant changes	[90,117]

## Data Availability

No new data were created or analyzed in this study.

## Abbreviations

The following abbreviations are used in this manuscript:

ETA	Endothelin receptor A
ETB	Endothelin receptor B
CP	Cardioplegia
CPB	Cardiopulmonary bypass
PKC	Protein kinase C
ECE	Endothelin-converting enzyme
GPCR	G protein-coupled receptor
PI3K	Phosphoinositide 3 kinase
TGFβ	Transforming growth factor beta
PLC	Phospholipase C
DAG	Diacylglycerol
IP3	Inositol triphosphate
ERK	Extracellular signal related kinase
